# Functional Characterization of Coat Protein and V2 Involved in Cell to Cell Movement of *Cotton Leaf Curl Kokhran Virus-Dabawali*

**DOI:** 10.1371/journal.pone.0026929

**Published:** 2011-11-16

**Authors:** C. G. Poornima Priyadarshini, M. V. Ambika, R. Tippeswamy, H. S. Savithri

**Affiliations:** Department of Biochemistry, Indian Institute of Science, Bangalore, India; Indian Institute of Science, India

## Abstract

The functional attributes of coat protein (CP) and V2 of the monopartite begomovirus, *Cotton leaf curl Kokhran virus- Dabawali* were analyzed *in vitro* and *in vivo* by their overexpression in *E coli,* insect cells and transient expression in the plant system. Purified recombinant V2 and CP proteins were shown to interact with each other using ELISA and surface plasmon resonance. Confocal microscopy of Sf21 cells expressing V2 and CP proteins revealed that V2 localized to the cell periphery and CP to the nucleus. Deletion of the N terminal nuclear localization signal of CP restricted its distribution to the cytoplasm. GFP-V2 and YFP-CP transiently expressed in *N.benthamiana* plants by agroinfiltration substantiated the localization of V2 to the cell periphery and CP predominantly to the nucleus. Interestingly, upon coinfiltration, CP was found both in the nucleus and in the cytoplasm along with V2. These results suggest that the interaction of V2 and CP may have important implications in the cell to cell movement.

## Introduction

Plant viruses are challenged by the presence of the “cell wall” and they need to traverse this barrier while moving from an infected cell to an adjacent cell. Hence, they employ the resident communication system, plasmodesmata (PD) which permit direct intercellular exchange of macromolecules [1], [2]. However, the PD openings are too small to permit passage of viral genomes or the viruses. Thus, the plant viruses encode one or more proteins, called movement proteins (MPs) that are essential for viral movement. MPs increase size exclusion limit [3], [4], interact with the endoplasmic reticulum and the cytoskeleton [5], [6] and also interact or modify diverse host factors to ensure successful spread [7], [8]. Most of the studies on viral movement are on RNA viruses, which replicate in the cytoplasm and can access the PD easily. However, DNA viruses replicate in the nucleus and have to cross the nuclear envelope to reach PD and subsequently move to the neighboring cell.

**Figure 1:**
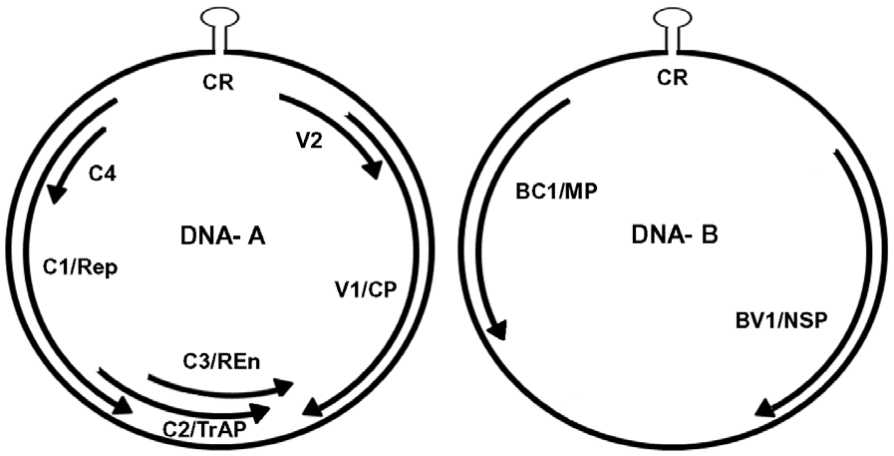
Typical genomic organization of begomoviruses. Open reading frames (ORFs) are denoted as either being encoded in the virion-sense (V) or complementary-sense (C) strand, preceded by component designation (A or B). The A component encodes V2; CP, coat protein; Rep, replication-associated protein; TrAP, transcriptional activator protein; REn, replication enhancer protein. The B component encodes NSP, nuclear shuttle protein and MP, movement protein; CR, common region.

Geminiviruses possess a small circular single stranded DNA (ssDNA) as their genome and are the causative agents for decreased yield in many economically important crops. They infect both monocotyledonous and dicotyledonous plants in tropical and subtropical regions [9]. Their genome is approximately 2.5–3.0 kb in size which is encapsidated in characteristic twinned particles, consisting of two incomplete T = 1 icosahedra [10]. Begomoviruses, a subgroup of geminiviruses are bipartite with two molecules of circular single stranded DNA (A and B), **Figure 1**. DNA-A encodes proteins that are essential for encapsidation and replication, DNA-B encodes nuclear shuttle protein (NSP or BV1) and movement protein (BC1 or MP) required for systemic spread [11].The viral DNA replicates via double stranded intermediate in the nuclei of infected plants [12]. NSP is essential for the transport of viral DNA across the nuclear envelope while MP is required for cell to cell movement through the PD [13]. However, the coat protein (CP) is shown to complement the function of NSP when disabled by mutations. [14].

Cotton leaf curl disease (CLCuD) causing viruses are monopartite begomoviruses having a single genome (DNA-A) and are often found to be associated with DNA-β and DNA 1 satellite molecules [15], [16]. These viruses lack BV1 and BC1 and hence DNA-A encoded proteins need to carry out their function. It has been suggested that V1, V2 and C4 could replace their function [17], [18], [19]. Gene disruption and mutational studies on *Tomato yellow leaf curl virus* (TYLCV) and *Tomato leaf curl virus* (TLCV) have shown that V1 (CP), could replace the function of NSP [18], [20]. Based on microinjection of *E. coli* expressed proteins and transient expression assays, Rojas *et.al.,* (2001) have proposed a model for TYLCV movement, in which CP mediates the nuclear export of double stranded DNA (dsDNA) for cell to cell and long distance movement within the plant. The export of DNA is further enhanced by CP at the nuclear periphery and the DNA is delivered to C4 at the cell periphery. C4, through its N-terminal myristoylation domain possibly mediates cell-to-cell transport via the PD. Further, V2 was found to be involved in viral spread [19], [20], in suppression of post-transcriptional gene silencing (PTGS) [21], virulence determination and in enhancing CP mediated nuclear export in *Tomato leaf curl Java virus-A* (ToLCJV-A) [22]. V2 was also shown to interact with host SGS3 to counteract the innate immune response of the host plant [23]. Co-inoculation experiments on *Tomato leaf curl New Delhi virus* (ToLCNDV) DNA-A and the DNA-β associated with CLCuD have shown that the βC1 is essential for the systemic infection. Further, the heterologous βC1 protein was shown to replace the movement function of the DNA-B of a bipartite begomovirus [24]. Notably all the studies on movement for monopartite begomoviruses are on viruses that cause leaf curl disease in tomato, and none are reported for viruses causing leaf curl disease in cotton. Furthermore, the function of V2 encoded by CLCuD causing viruses remains unclear [25].

We have reported earlier the DNA-A sequences of CLCuD causing monopartite begomoviruses and demonstrated the genetic diversity of begomoviruses associated with cotton leaf curl disease in India [26]. CP was shown to interact with DNA via the N terminal zinc finger motif and H85 of this motif was shown to be the most important residue for DNA binding [27]. In the present investigation, we show that the V2 and CP of *Cotton Leaf curl Kokhran virus-Dabawali* (CLCuKV-Dab) interact with each other using ELISA and Surface Plasmon Resonance (SPR). Transient expression of these proteins in insect cells and in *N. benthamiana* showed that CP localized to nucleus whereas V2 localized to cell periphery. Coinfiltration studies in plants revealed that CP is in the cytoplasm along with V2, which suggests that they may interact with each other and play a predominant role in viral cell to cell movement. A model for cell to cell movement of CLCuKV-Dab is proposed based on these results.

## Results and Discussion

### Expression and purification of V2 and GST-CP

The V2 gene was cloned and overexpressed in *E.coli* as described in materials and methods. The protein was found to be soluble only when overexpressed in *E.coli* Origami (DE3) strain at a lower temperature (20°C). The soluble protein was purified using Ni-NTA affinity chromatography. However, many recombinant viral MPs expressed in *E. coli* are reported to form inclusion bodies that hamper their biochemical and biophysical characterization [28], [29].

**Figure 2:**
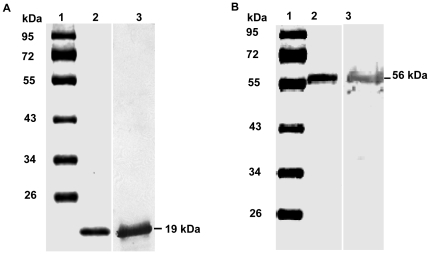
Purification of V2 and GST-CP. V2 and GST-CP genes were overexpressed in *E.coli* and purified using Ni-NTA and GSH agarose affinity chromatography respectively. The purified protein was analyzed on 12% SDS-PAGE and stained with coomassie R250. Lane 1, protein molecular mass markers. Lane 2, purified recombinant V2 which migrated as a single band at ∼19 kDa (**A**) and GST-CP fusion protein of Molecular mass 56 kDa (**B**). Western blot analysis of purified V2 and GST-CP performed using polyclonal antibodies to V2 and GSTCP respectively (Lane 3, A&B).

Similarly, the CP was overexpressed as GST fusion protein and purified as described in the methods section. The purified V2 and GST-CP were analyzed by SDS PAGE **(** **Figure 2A&B** **lane 2 respectively)** and western blotting using anti-V2 and anti GST-CP antibodies **(** **Figure 2A&B** **lane 3 respectively)**.

### CLCuKV-Dab V2 has primarily α-Helical Structure

**Figure 3:**
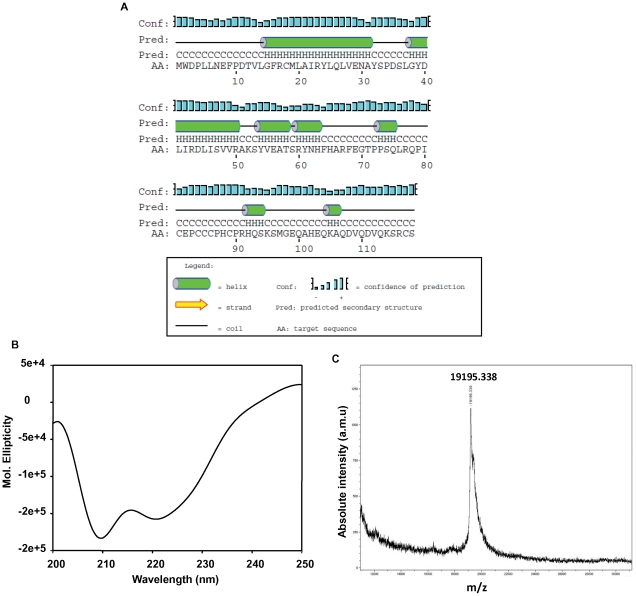
Biophysical characterization of recombinant V2. (A) Secondary structure analysis of CLCuKV-Dab V2. Graphical output of consensus secondary structure prediction of V2 generated using the PSIPRED web server, confidence scores (conf) are also shown. (**B**) Far-UV circular dichroism (CD) spectrum of V2 (0.5 mg/ml) in 50 mM Tris, pH 8.0 at 25°C. (**C**) Molecular mass of purified V2 was assessed by MALDI mass spectrometry performed using a Ultraflex MALDI TOF/TOF mass spectrometer fitted with a standard 337 nm nitrogen laser.

A secondary structure prediction was carried out on the amino acid sequence of V2 using the PSIPRED Protein Structure Prediction Server [30], [31]. The results predicted that the protein may have substantial α-helical structure **(** **Figure 3A** **)**. This was confirmed experimentally by far-UV CD analysis of purified recombinant V2, which showed the minima at 209 and 222, indicating that the protein is folded and adopts a largely α-helical conformation **(** **Figure 3B** **)**. The expected molecular mass of the V2 protein was 19000 Da and it was further confirmed by mass spectrometry to be 1915.338 Da **(** **Figure 3C** **)**. We have recently shown that *Sesbania Mosaic Virus* MP is also a helical protein which interacts with its CP [32]. Yet another helical protein well characterized for its role in movement is *Tobacco mosaic virus* (TMV) MP [29].

### V2 interacts with CP *in vitro*

**Figure 4:**
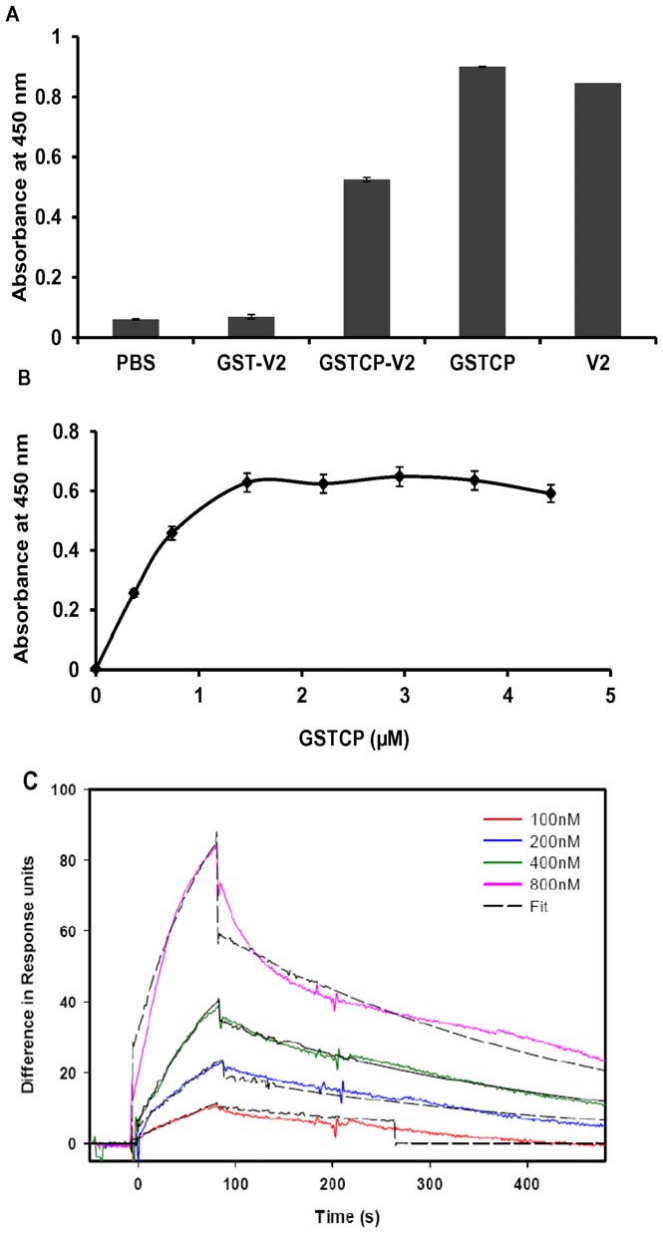
V2 and CP interact in a concentration dependent manner. ELISA and SPR based protein-protein interaction studies were performed. In both the cases, V2 was coated onto matrix. (**A**) ELISA. The wells of micro titer plate were coated with PBS, GST, GST-CP and V2. Bar PBS, well coated with PBS, allowed to interact with V2 and probed with V2 antibodies. Bar GST-V2, well coated with GST and the interacting protein V2 was probed with V2 antibodies. Bar GST-CP-V2, same as the previous experiment except GST-CP was used instead of GST. Bar V2 well coated with V2 and probed with V2 specific antibodies (direct antigen coating ELISA) Bar GST-CP well coated with GST-CP and probed with GST-CP specific antibodies (direct antigen coating ELISA). (**B**) V2 coated wells were probed with increasing concentrations of GST-CP (0–5 µM). Interaction was scored using anti GST-CP antibodies (primary antibodies), secondary antibody conjugated to HRP and the absorbance measured at 450 nm. The absorbance values represent average of three independent experiments and vertical bar represents the standard error. (**C**) SPR. Sensorgrams of the interaction between V2 and GST-CP indicate the phases of association (after GST-CP addition) and of dissociation (after exposure of the chip to buffer). Various concentrations of GST CP (100–800 nM) were passed over the chip containing 340 RU of V2. The experiment was performed at a flow rate of 20 µl min^−1^, allowing 90 s of association and 350 s of dissociation. X-axis represents the time in seconds (s) and Y-axis the difference in response units (RU). The fit for the recorded sensorgrams are shown as black discontinuous lines. Injections were carried out in duplicates, which gave essentially the same results. Only one of the duplicates is shown.

In order to understand the mechanism of movement of CLCuKV-Dab and the role of CP and V2 in the process, we performed direct interaction studies *in vitro* by an ELISA based assay. Purified V2 or GST-CP was coated onto ELISA plates and incubated with increasing concentrations of interacting protein. The V2 and GST-CP interaction was assessed by either anti V2 or anti GST-CP specific antibodies. As shown in **Figure 4A**, V2 was found to interact with GST-CP. The interaction was specific as there was no absorbance observed either for GST or for the buffer control. Direct ELISA with V2 and GST-CP proteins with their specific antibodies was also performed in parallel as positive controls **(** **Figure 4A** **)**. The interaction was further found to be concentration dependent **(** **Figure 4B** **)**.

### Surface Plasmon Resonance (SPR) studies

The V2-CP interaction was quantified using SPR. The V2 was immobilized on the Ni-NTA chip and the experiments were performed as described in the methods section. **Figure 4C** depicts the sensorgrams obtained for the binding of GST-CP to V2. The response from the control surface (buffer alone) was subtracted from the V2 immobilized surface and the relative response (in response units, RU) was plotted as a function of time to obtain the association and dissociation constants for GST-CP and V2 interaction. The binding curves at various concentrations of GST-CP indicated that the binding of GST-CP to V2 was dose-dependent **(** **Figure 4C** **)**. The kinetic constants were determined using BIA evaluation software 3.0. The global fitting analyzes both association and dissociation data for all concentrations simultaneously using a 1∶1 Langmuir binding model. A random distribution of residuals and a χ^2^ value for this interaction indicated that this model describes well the experimental data. The estimated *k_a_* and *k_d_* values of the interaction are 1.03×10^3^ (M^–1^s^–1^) and 2.67×10^–3^ (s^–1^), respectively. The *K_D_* value was calculated to be of 2.6×^–6^ M. Thus our results clearly demonstrate the direct interaction of CLCuKV-Dab V2 with CP. The interaction of proteins encoded by the viral genome with each other and with many other host proteins [12], [33] is crucial for successful infection. It was shown earlier that the CP of *Maize streak virus*, a geminivirus that infects monocots, interacts with its MP [34]. Further, BV1 (NSP) and BC1 (MP) of a begomovirus, *Squash leaf curl virus* (SqLCV) was shown to interact cooperatively [35]. However, in the case of AbMV, yeast two hybrid analysis revealed that, the two proteins do not interact [36]. Thus there are contradicting reports on NSP and MP physical interaction to transport the viral DNA to the neighboring cell (reviewed in Rojas et al. 2005). NSP is also reported to interact with several host factors, such as PERK like receptor kinases, [37], acetyltransferase AtNSI [38] and protein kinase like kinase [39]. ToLCV V1 interacts with a host factor SlUPTG1, which appears to play an important role in infection [40].

### Localization of V2 and CP in insect cells

#### V2 localizes to cytoplasm and cell periphery

**Figure 5:**
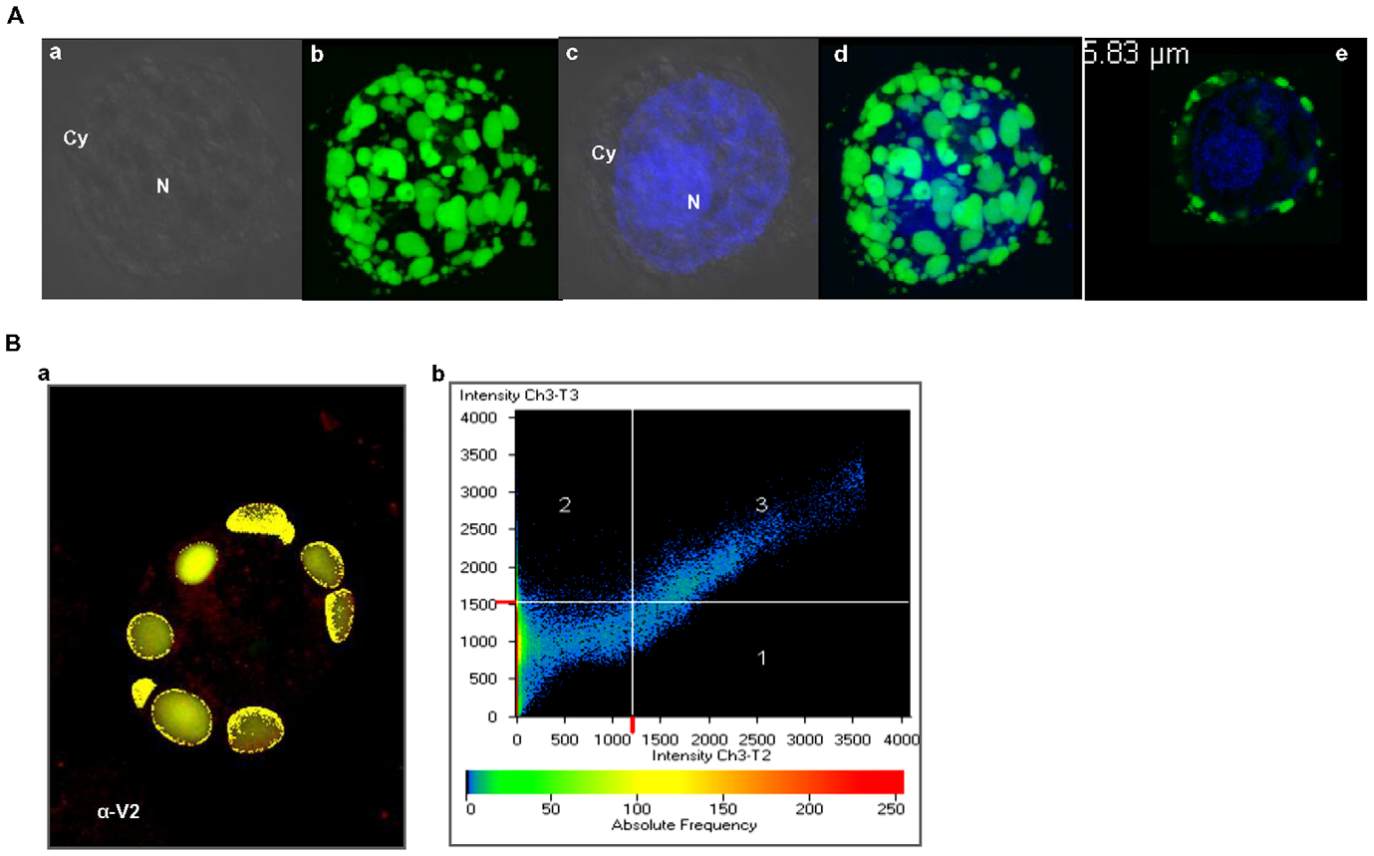
Localization of baculovirus-expressed GFP-V2 in Sf21 cells. Representative images recorded at 60 h post baculoviral infection using confocal microscopy. The green is due to fluorescence of GFP or of GFP fusion proteins and blue stain (DAPI) is for the nucleus. (N, Nucleus; Cy, cytoplasm). (**A**) GFP-V2 localization (a) Bright field image of GFP-V2 expressing cell. (b) GFP expression at the cytoplasm/cell periphery (c) nuclear staining using DAPI (d) shows the merged image of b and c confirming the distribution of V2 outside the nucleus. Panel (e) shows the representative Z section of a cell expressing V2. Number in white represents the depth of the sections. (**B**) Immunolocalization studies of V2. Sf21 cells expressing GFP-V2 were probed with rabbit anti-V2 antibodies, followed by incubation with a TRITC-conjugated anti-rabbit IgG secondary antibody and both green and red fluorescence were detected using different channels. Green (GFP) and Red (TRITC) colocalized pixels were superimposed in (a) and they correspond to region 3 of the scattergram (*b*). Scattergrams show the pixel intensity distribution for the fluorescence of each Fluor (488 and 568). The crosshair lines in the scattergrams were positioned above the calculated background threshold for each Fluor. The crosshair lines define four regions: region 1 corresponds to TRITC pixels only; region 2 corresponds to GFP pixels only; region 3 contains the pixels where the GFP–TRIC overlap is the greatest; region 4 corresponds to sub-threshold pixels. Co-localization analysis was performed with Carl Zeiss LSM 5 software.

The localization of V2 was monitored by observing the GFP localization in the GFP-V2 fusion protein. Sf21 cells were infected with recombinant baculovirus expressing GFP-V2 as described in the methods section. The cells were fixed, stained for nucleus with DAPI and observed under confocal microscope. The GFP-V2 expressing Sf21 cell and the DAPI staining of the same cell **(** **Figure 5A** **a–d)** showed that the V2 distribution is outside the nucleus. Z-sections were taken at every 0.36 µm and a representative Z section image is shown in **Figure 5A** **(e)**. The GFP fluorescence was predominantly seen as punctuate bodies in the cytoplasm and at the cell periphery. In contrast, GFP alone was uniformly distributed throughout the cell **(Figure S1)**. To further confirm that the localization of GFP fusion protein is indeed due to V2 moiety, immunocolocalization studies were performed scoring for both GFP and V2 expression individually. V2 was detected by indirect immunofluorescence using V2 specific antibodies and TRITC-conjugated secondary antibodies. The degree of colocalization was measured by plotting the scattergram as described in methods section. The colocalized pixels are located along the diagonal, while those with no colocalization occupy left and bottom portions **(** **Figure 5B** **)**. For V2, colocalization and overlap coefficients of 0.76 and 1.0 were obtained, respectively. The overlap coefficient of 1 indicates significant colocalization. The corresponding colocalization percentage was calculated to be 60%.

### CP is predominantly found in the nucleus

**Figure 6:**
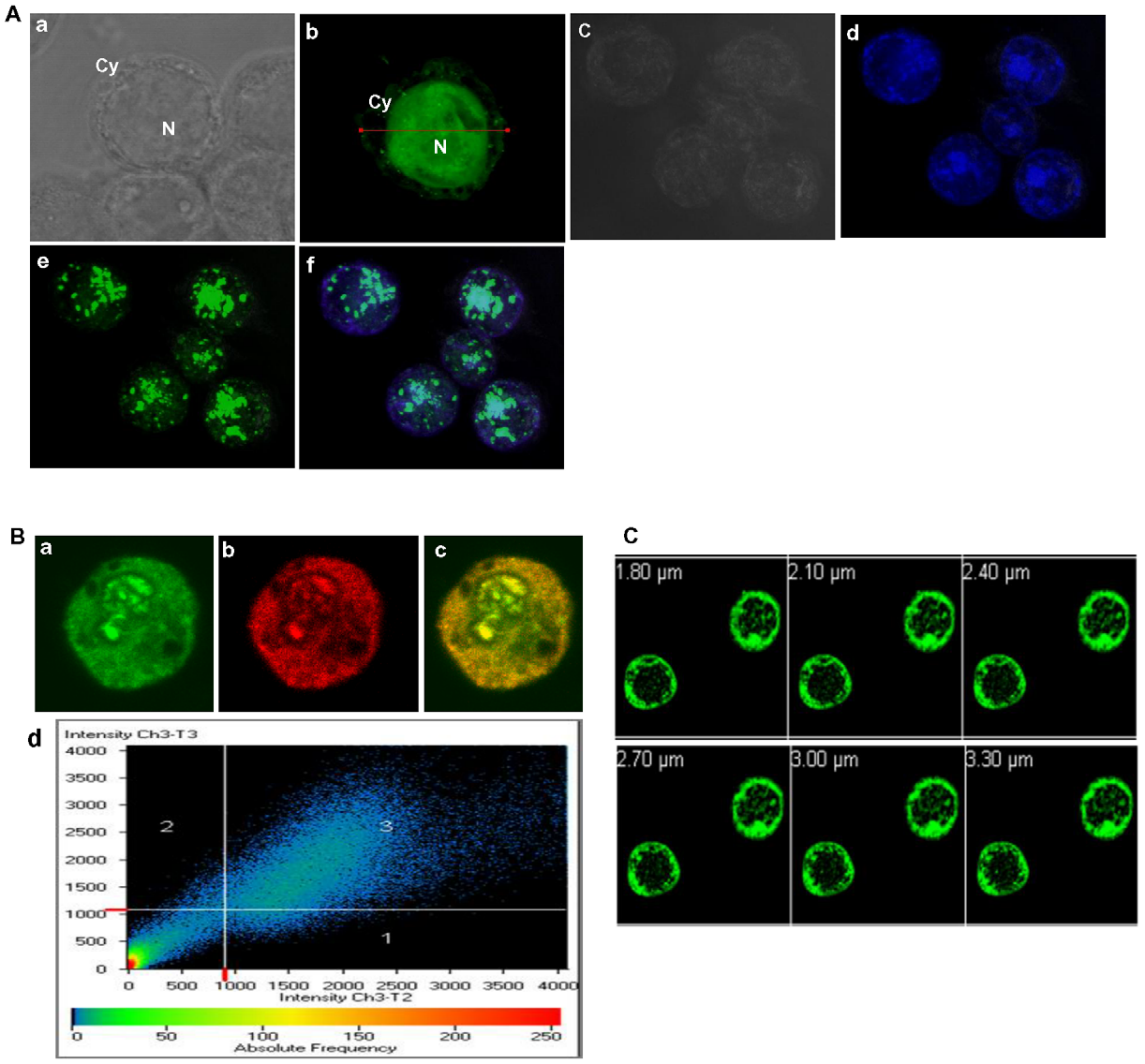
Localization of CP in Sf21 cells. (**A**) 3D projected image of the representative cell expressing GFP-CP showing the uniform distribution of CP in the nucleus after 60 hr. post baculoviral infection (a) bright field image and (b) GFP-CP expression was observed monitoring GFP. Images d–f, show the discrete bodies formed at a later period of post baculoviral infection (72hr.p.i). DAPI staining for the nucleus (d) and the merge of both d & e confirms the presence of CP in the nucleus (f). (**B**) Sf21 cells expressing GFP-CP were probed with rabbit anti CP antibodies and detected using TRITC conjugated anti rabbit IgG secondary antibodies. GFP (a) and TRIC (b) colocalized pixels were superimposed (c) and correspond to region 3 of the scattergram (d). Scattergrams show the pixel intensity distribution for the fluorescence of each Fluor (488 and 568). The crosshair lines in the scattergrams were positioned above the calculated background threshold for each Fluor. The crosshair lines define four regions: region 1 corresponds to TRITC pixels only; region 2 corresponds to GFP pixels only; region 3 contains the pixels where the GFP–TRIC overlap is the greatest; region 4 corresponds to sub-threshold pixels. Co-localization analysis was performed with Carl Zeiss LSM 5 software. (**C**) Expression pattern of GFP-CP NLS deletion mutant. The Sf21 cells expressing NLS deletion mutant was observed under the confocal microscope. Z sections of the cells indicating the distribution of the CP restricted to the cytoplasm. Number in white represents the depth of the sections.

As shown in **Figure 6A**, the distribution of GFP-CP was predominantly in the nucleus **(b)**. Initially, the distribution of CP was uniform in the nucleus (b) but, at later periods of baculoviral infection, CP was found to accumulate in the nucleus as discrete bodies (c–f). Like in V2, immunocolocalization studies were carried out to confirm that the localization of GFP-CP fusion protein is due to CP moiety using CP specific primary and TRITC-conjugated secondary antibodies. Clear merge (c) of the fluorescence due to GFP (a) and TRITC (b) and the scattergram (d) confirm the CP localization in the nucleus **(** **Figure 6B** **)**. Colocalization parameters were calculated for CP as described earlier. The colocalization and overlap coefficients of 0.98 and 1.0 respectively, were obtained. Like in V2, the overlap coefficient was 1 indicating significant colocalization. The corresponding colocalization percentage was calculated to be 92%.

Bioinformatic analysis of CP by Expasy ScanProsite (http://expasy.org/tools/scanprosite/) and PSORT (http://psort.nibb.ac.jp) tools predicted a putative bipartite localization signal, ^1^MS***KR***PADIIISTPAS***K***V***RRR***INF ^23^ at the N terminus of CP. When the amino acid sequence corresponding to the bipartite nuclear localization signal was deleted, the expression of CP was restricted to the cytoplasm substantiating the importance of the signal sequence for nuclear localization of CP **(** **Figure 6C** **)**. Interestingly, the NET- program [41], [42] revealed that the CP also has a nuclear export signal (NES) motif at the C-terminal end suggesting that CP could function as a nuclear shuttle protein. Analysis of the NLS and NES sequences of many representative CPs of begomoviruses revealed that they were conserved across this genus **(Figure S2).**

### Transient expression of V2 in plants

**Figure 7:**
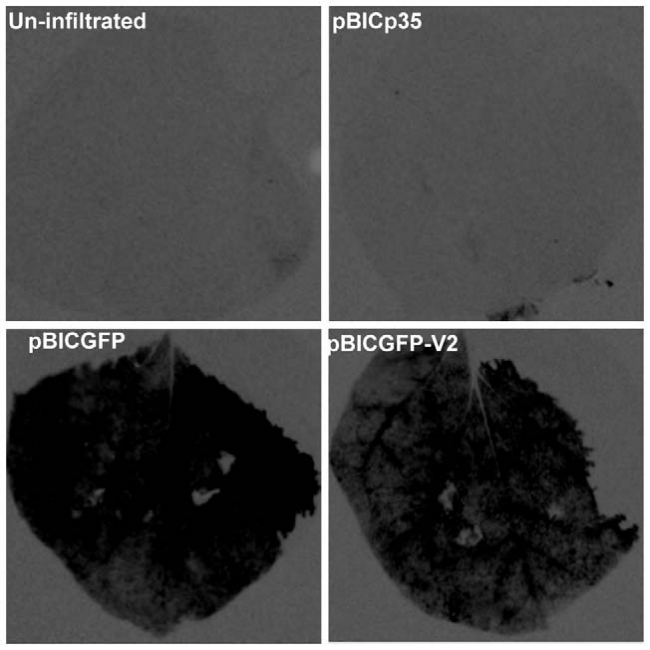
Whole leaf fluorescence observed under LAS 3000 imager. EHA 105 cells transformed with pBIC GFP and pBIC GFP-V2 were infiltrated into *N. benthamiana* as described in materials and methods and the whole leaf is observed under LAS 3000 imager 60 hours post infiltration. Fluorescence was observed throughout the leaf infiltrated with pBIC GFP and pBIC GFP-V2. In contrast no fluorescence was observed in the leaf infiltrated with EHA 105 transformed with empty vector and in uninfiltrated leaves.

With the preliminary information on localization of V2 and CP in insect cells, we validated our observations in the plant system. The pBIC constructs were transformed into agrobacterium strain EHA 105 and the transformed cells were infiltrated into *N. benthamiana* leaves as described in the methods section. Agroinfiltrated leaves were harvested 60 hours post infiltration. GFP fluorescence was visualized under Fujifilm LAS 3000 imager. As shown in the **Figure 7**, the fluorescence was observed throughout the leaf when GFP and GFP-V2 were expressed as against the uninfiltrated leaves and in the leaves infiltrated with EHA 105 transformed with empty vector.

### Intracellular localization of CP and V2 in plants

#### V2 localizes to cell periphery

**Figure 8:**
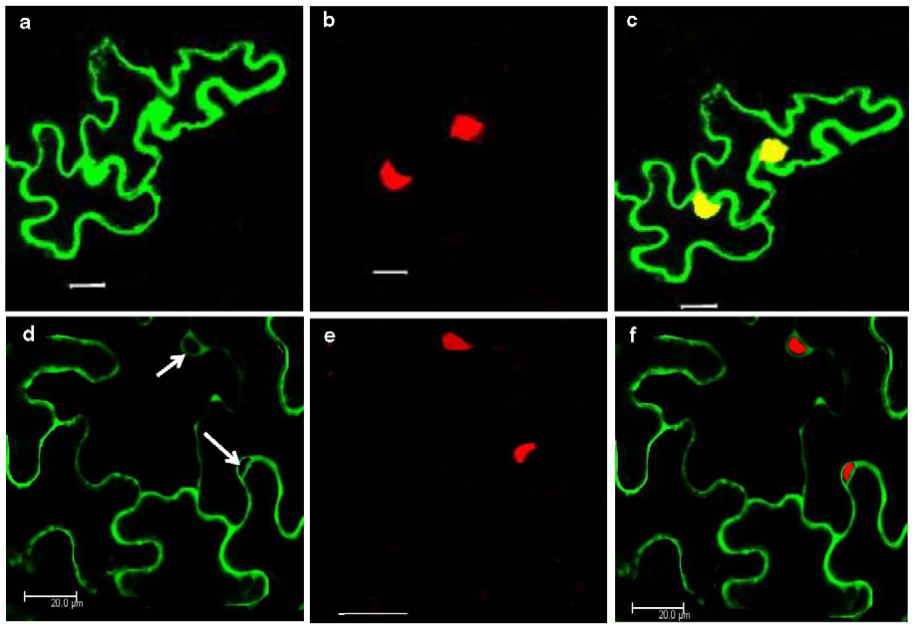
Subcellular localization of CLCuKV-Dab V2 fusion protein in *N.benthamiana.* Confocal microscopy of leaves infiltrated with EHA 105 harboring pBICP35GFP and pBICP35GFP-V2 60 hours post infiltration. (**a**) GFP alone fluorescence dispersed throughout the cell (**b**) Propidium iodide staining was restricted to nucleus. (**c**) Merged imaged of (a) and (c). (d) GFP-V2 localized to perinuclear region and cell periphery 60 hours post infiltration. Arrows indicate the perinuclear region. (**f**) The overlay of (d) with Propidium Iodide nuclear staining (e) ratifies the absence of V2 in the nucleus. Bar  = 20 µm

The subcellular localization of V2 in *N.benthamiana* was visualized by confocal microscopy as described in the methods section. When GFP was expressed alone, there was uniform distribution throughout the cell that is in the nucleus and the cytoplasm **(** **Figure 8** **a–c)**. A large part of the mature leaf cell is occupied by the vacuole and due to which the cytoplasmic space appears as lining adjacent to the plasma membrane. Nucleus is stained using Propidium Iodide, PI **(** **Figure 8** **b & e)**. GFP-V2 remained primarily in the cell periphery, although its presence in the cytoplasm could not be ruled out **(** **Figure 8** **d & f)**. Similar results were obtained with TYLCV [19] and ToLCV [40]. TYLCV V1-GFP was distributed around the nuclear periphery and to the cell periphery. It was also demonstrated that V1 was able to increase the size exclusion limit of plasmodesmata in a low proportion of cells. The study further suggested the interaction of V1 with endoplasmic reticulum network [19]. In ToLCV, V1-GFP was targeted to the cell periphery as punctuate fluorescent spots that indicated plasmodesmal localization particularly in plasmolysed cells [40]. This is also in accordance with the results presented in this paper and those obtained with *Tomato leaf curl Java virus-A* (ToLCJV-A), where V2 was shown to localize to nuclear periphery and cell periphery [43].

### CP localizes to nucleus

**Figure 9:**
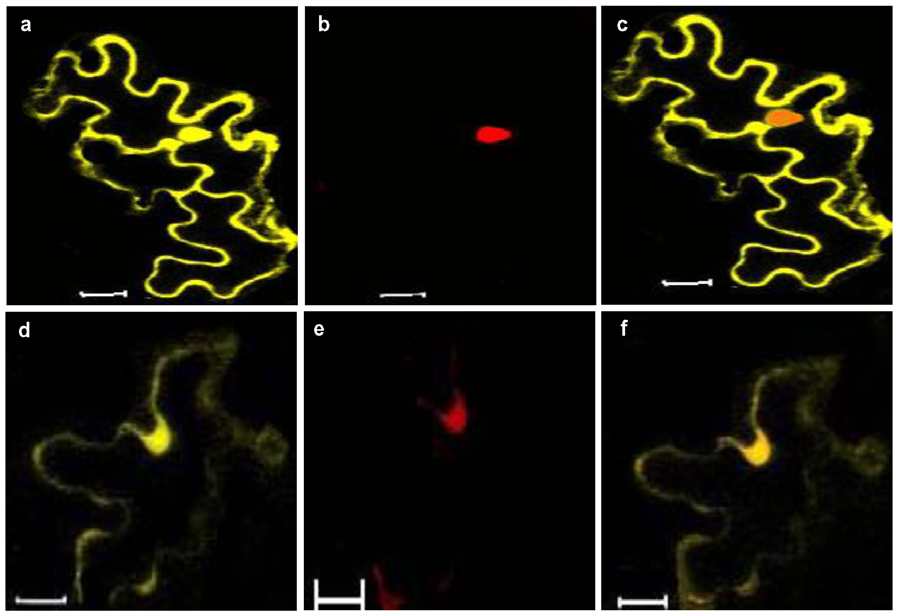
Subcellular localization of CLCuKV-Dab CP fusion protein in *N.benthamiana.* (**a**) YFP alone, Propidium iodide staining restricted to nucleus (**b**) and the (**c**) Merged imaged of (a) and (c) shows the YFP distribution throughout the cell. YFP-CP fusion protein was targeted to nucleus (**d**). (**e**) Propidium iodide staining of the nucleus. The overlay of (d) and (e) correlates with the presence of CP in the nucleus (**f**). Bar  = 20 µm

The confocal image of YFP-CP depicts that CP is limited to the nucleus. This was confirmed by PI staining which was restricted to the nucleus **(** **Figure 9** **d**–**f)**. In contrast, YFP alone appears to be distributed throughout the cell **(** **Figure 9** **a**–**c)**. The fact that CP localized to the nucleus even in the absence of genomic DNA suggests that during the life cycle of the virus the CP that is translated in the cytoplasm can enter the nucleus and once in the nucleus it could specifically interact with genomic ssDNA. We have shown earlier that ToLCBV-Ban5 CP [44] as well as CLCuKV-Dab CP [27] bind preferentially to ssDNA.

### Colocalization studies of CP and V2

**Figure 10:**
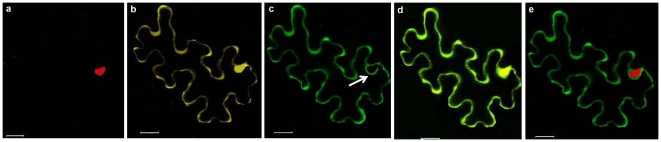
Coinfiltration profile of YFP-CP and GFP-V2 constructs. (**a**) Propidium iodide staining of nucleus. (**b**) YFP-CP fusion protein localized to nucleus and cell periphery. (**c**) GFP-V2 fusion protein was targeted to perinuclear region (shown by arrows) and cell periphery. (**d**) Overlay of b and c confirms the presence of CP in cytoplasm along with V2. (**e**) Overlay of (a) and (c) confirms the absence of V2 in the nucleus. Bar  = 20 µm

As CP and V2 interact with each other *in vitro* as demonstrated by ELISA and SPR studies, it was of interest to examine the localization of these two proteins when expressed together. Therefore, agrobacterium harboring the CP and V2 constructs were coinfiltrated into *N. benthamiana* plants. As shown in the **Figure 10**, the individual **(b & c)**, and merged **(d)** confocal images of CP and V2, and the co-staining of nucleus with PI **(a)** unveiled that CP was found in the nucleus as well as in the cytoplasm along with V2. V2 was also localized to the cell periphery and nuclear periphery **(** **Figure 10** **b & e)**. Thus the coinfiltration of CLCuKV-Dab V2 and CP transformants revealed that the localization pattern of CP is altered. These results suggest that the interaction of nuclear localized CP with V2 present at the nuclear periphery might render the complex to move out of the nucleus and hence both proteins are seen in the cytoplasm. Similarly, transient expression studies have shown that AbMV MP can redirect movement of NSP from the nucleus [45]. Recent findings have demonstrated the association of DNA-β in viral movement and pathogenicity. Localization and interaction studies of BYVMD CP and βC1 [46] and together with studies of [24] suggest the possible role of these proteins in the cell-to-cell movement of virus. However, the precise role of βC1 in cell-to-cell movement needs to be dissected.

### Conclusion

Based on the results presented in this paper, a model for cell to cell movement of CLCuKV-Dab is proposed. The CP translated in the cytoplasm is targeted to the nucleus via its NLS, where it binds to progeny ssDNA and exports the ssDNA out of nucleus acting as a nuclear shuttle protein. V2 present at the nuclear periphery might interact with CP-DNA complex and assist in the nuclear export. The complex together with V2 might be transported to the cell periphery via the interaction of other viral encoded protein such as C4 and other host factors. The complex is then transported from one cell to the neighboring cell via PD. Both C4 and V2 in addition to being involved in movement function have been shown to act as suppressors of gene silencing and as pathogenicity factors [21], [47], [48]. The latter two functions could represent disruption of the two arms of the defense/development mechanism one involving siRNA and the other miRNA. Studies are in progress to probe if V2 and C4 together modulate these functions.

## Materials and Methods

All the *E.coli* strains used in the study and the affinity purification kits were purchased from Novagen–EMD4Biosciences (USA). Baculovirus expression system was purchased from Invitrogen, Life technologies, (USA).The chemicals and the secondary antibodies were procured from Sigma-Aldrich (USA). The primary polyclonal antibodies used in the study were raised in our laboratory.

### Cloning and expression of CLCuKV-Dab V2 and CP

#### E coli

The total gDNA was isolated from the CLCuKV-Dab infected cotton leaf material and used as template DNA to amplify V2 gene by PCR with gene specific primers: forward primer for V2; 5’ CATGCCATG*GCTAGC*TGGGATCCACTGTTAAATG 3’ and reverse primer for V2; 5’ CGGAATTCTTACTCGAGGGAACATCTGGACTTC 3’. V2 was cloned into the pRSET C at *PvuII* site in order to obtain a hexahistidine tag at its N-terminus. The CP gene was PCR amplified from pRCP clone [27] using forward primer; 5'CATGCCATG*GCTAGC*TCGAAGCGACCAGC 3' and reverse primer; 5’ CG*GGATCC*TTACTCGAGATTTGTCACGGAATC 3'. CP gene was then cloned into pGEX-5X-2 vector (Novagen) at *SmaI* site and there by a GST tag at the N-terminus of CP was fused. Both the clones were confirmed by DNA sequencing.

The pRSETC V2 (pR-V2) and pGEX-CP plasmids were transformed into *E.coli* strain Origami™ B (DE3) and BL21 (DE3) pLysS respectively and the proteins were overexpressed as indicated in the manufacturer's instructions (Novagen). Both the overexpressed proteins were soluble when the cultures were grown initially at 30°C and at 20°C post induction.

### Baculovirus constructs

**Figure 11:**
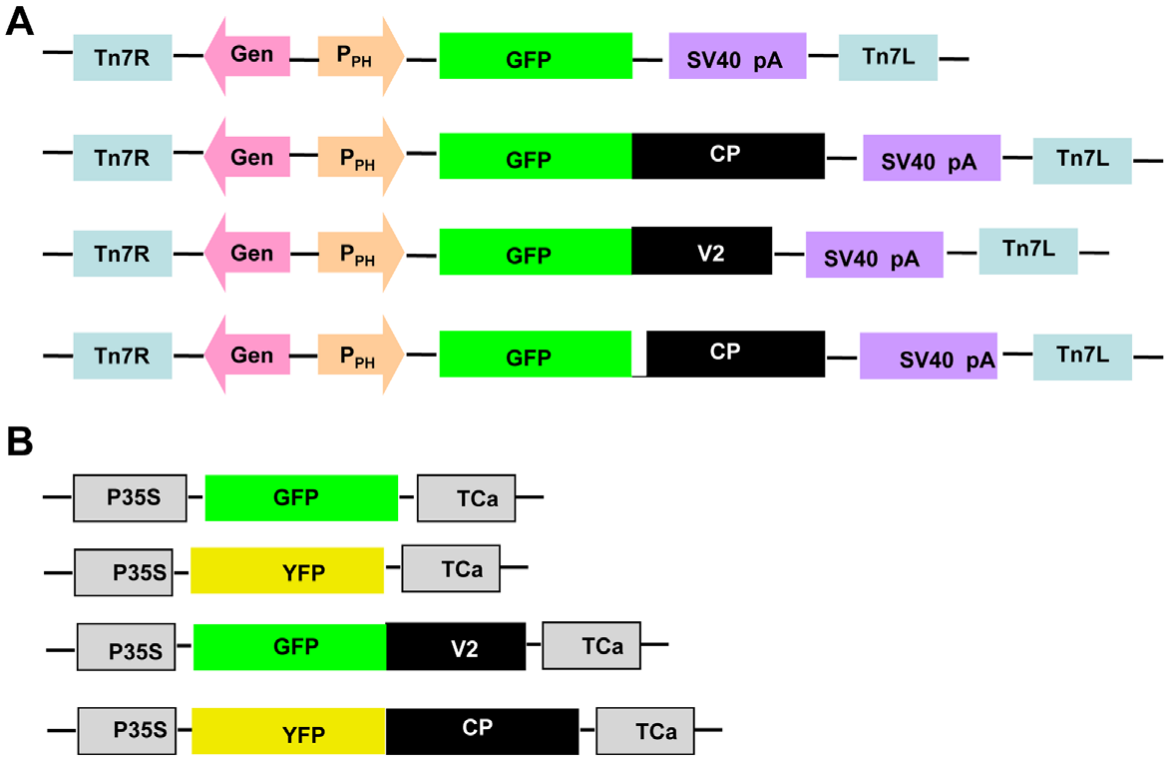
Constructs for localization studies in insect cell lines and plants. (**A**) Partial map of pFAST Bac vector containing GFP, GFP-CP, GFP-CP del NLS and GFP-V2. The expression of these genes are controlled by the *Autographa californica* multiple nuclear polyhedrosis virus (AcMNPV) polyhedrin (PH) promoter for high-level expression in insect cells. The expression cassette is flanked by the left and right arms of Tn7, and a gentamicin resistance gene and a SV40 polyadenylation signal. (**B**) Partial map of pBIC P35 binary vector containing GFP, YFP, GFP-V2 and YFP-CP. P35S and TCa corresponds to 35S promoter and terminator from Cauliflower mosaic virus respectively.

GFP, GFP-V2 GFP-CP and GFP-CP del NLS (CP in which the nuclear localization signal NLS was deleted) were cloned in a donor vector, pFastBac1 (Invitrogen, Life technologies) individually under Polyhedrin promoter **(** **Figure 11A** **)**. The GFP fusion was at the N-terminus of the V2 and CP. The GFP-V2 and GFP-CP fusion constructs were generated by inserting a restriction site *Stu1* and a linker sequence corresponding to a stretch of serine and glycine residues in the GFP antisense primer. The primers used were as follows. Forward primer: 5’-ATGGATCCCCAGGTACCGGTCGCCACCATAGTG 3’; reverse primer: 5′-*AGGCCT*TCCGGAGGAGGACTTGTACAGCTCGTCC 3’; the restriction site for *Stu1* is underlined. All the clones were confirmed by sequencing **(** **Figure 11A** **)**.

### Binary constructs

GFP, YFP (Yellow Fluorescent Protein), GFP-V2 and YFP-CP were cloned into binary vector pBICP35. GFP was amplified from pEAQGFP vector and YFP from pDH5YFP vector and were inserted into *Stu1* site of pBICP35 vector. V2 and CP were fused to the C terminal of GFP and YFP respectively by cloning at *Kpn1* site. The primers used were as follows YFP forward primer: 5’GCCAGTAAAGGAGAAGAACTTTTCACT 3’ and YFP reverse primer: 5’GCCTCTAGAGTCTCCGGCTGGTCCGCCTCCTTCT 3’. All the clones were confirmed by sequencing **(** **Figure 11B** **)**.

### Protein expression in plants

Clones of interest were mobilized into *Agrobacterium tumefaciens* strain EHA105 by electroporation. Transformants were selected on 25 mg/ml Rifampicin and 50 mg/ml Kanamycin plates. Agroinfiltration was done as described elsewhere [49] with a few modifications. Briefly, cultures harboring each plasmid were grown overnight at 30°C from single colonies in LB broth containing Rifampicin-kanamycin, 10 mM MES (Morpholine Morph line Ethanesulfonic Acid pH 5.9) and 50 µM Acetosyringone. The cultures were centrifuged at 6,000 rpm for 15 min, and washed thrice with milli Q water. The pellets were resuspended in the infiltration medium (10 mM MgCl_2_, 10 mM MES, pH 5.9, and 150 µM Acetosyringone) and incubated at room temperature for a minimum of 3 to 5 hours. Bacterial cultures (at an optical density of 0.5 at 600 nm) were infiltrated by gently pressing the end of a 3-ml syringe loaded with appropriate culture to the leaf and exerting gentle pressure to flood the interstitial areas within the leaf.

### Protein purification

#### His-tagged V2

The IPTG induced *E.coli* cells were harvested and re-suspended in buffer (50 mM Tris, pH 8, 200 mM NaCl, 10% Glycerol, 1% Triton X-100) and lysed by sonication. The cell lysate was then spun down at 10,000 rpm for 10 minutes. The supernatant was used for purification of V2 using His-Bind resin (Novagen), according to the manufacturer's instructions.

### GST-CP fusion protein

GST and GST-CP were purified as described in the instruction manual (Novagen). In brief, culture supernatant obtained after sonication was mixed with glutathione sepharose (GSH) beads, pre-equilibrated with extraction buffer (1x Phosphate Buffered Saline (PBS) pH 7.4, 1 mM DTT and 0.1% Triton X-100) and incubated for 2 h at 4°C to allow the protein to bind to the beads. The beads were then packed in a column and washed with 50 and 20 bed volumes each of washing buffers I (1x PBS pH 7.4 and 1 mM DTT) and II (50 mM Tris-HCl pH 7.5 containing 1 mM DTT and 0.1% Triton-X 100) respectively. GST-CP was eluted with 50 mM Tris HCl pH 8.0, 200 mM NaCl and 20 mM Glutathione (reduced). The eluted fractions were checked for the presence of the protein on SDS-PAGE and by western blotting using anti-GSTCP antibodies.

### Cell culture, DNA transfection, baculoviral infection

*Spodoptera frugiperda,* Sf-21 cell lines (Invitrogen Corp.) were cultured at 27°C in insect cell medium (TC100, Sigma) with 10% serum supplement (Serum-plus, JRH Biosciences). Recombinant baculoviruses were transfected using cellfectin and propagated in Sf21 cell lines as indicated in the instruction manual.

### Enzyme-linked immunosorbent assay (ELISA) based binding studies

Interaction of V2 with CP was tested by ELISA as described previously with minor modifications using either GSTCP or V2 specific polyclonal antibodies [50], [51]. Five µg/well of purified V2, GST and GST-CP along with PBS control for **Figure 5A** and V2 alone for **Figure 5B** was coated onto wells of ELISA plates (Nunc Axisorp F96 F) and incubated overnight at 4°C. The wells were blocked with 5% skimmed milk in 1X PBS for 1 h at 37°C. The plates were then incubated with the interacting proteins for 2 h at room temperature (RT). The bound protein was detected by its spectific polyclonal antibodies, followed by goat anti rabbit secondary antibody conjugated to HRP. TMB/H_2_O_2_ was used as substrate. Wells were washed three times with PBS (pH 7.2) and PBST (containing 0.05% (v/v) Triton X-100) between incubations. Interactions were quantified by reading the absorbance at 450 nm using a Spectramass 340PC ELISA reader (Molecular devices). All the experiments were done in triplicates and standard deviation was calculated.

### Surface Plasmon Resonance

The binding kinetics of V2 and CP was determined by SPR using the BIAcore 2000 optical biosensor (GE Healthcare Lifescience, Uppsala, Sweden) operated at 25°C. The Nitrilotriacetic acid chip (NTA) was first saturated with Ni^2+^ by washing it with 500 µM NiCl_2_ (20 µl at 20 µl/min) followed by immobilization of purified His-tagged V2 (2 µl/min) up to 350 response units (RU) in eluent buffer. Unbound V2 was removed by passing the buffer at the flow rate of 100 µl/min. The binding reactions were carried out in a continuous flow of running buffer A (10 mM HEPES buffer pH 7.4, containing 150 mM NaCl, 50 µM EDTA and 0.005% surfactant P-20). The buffer for the sample pump was dispenser buffer (10 mM HEPES pH 7.4, 150 mM NaCl, 3 mM EDTA, 0.005% surfactant P-20). Regeneration was achieved by washing the flow cell with regeneration solution (10 mM HEPES pH 8.3, 150 mM NaCl, 3 mM EDTA, 0.005% Surfactant P-20). Various concentrations of GST-CP (100 nM– 800 nM) in the running buffer were injected at a flow rate of 20μl/min and the interaction was monitored for 90 sec. Dissociation was achieved with the running buffer containing 1 M NaCl. The specific changes in the experimental sensorgram were measured by subtracting the values of the reference cell containing no protein. The binding data were analyzed using a 1∶1 Langmuir binding model in BIAcore evaluation software, version 3.0

### Circular Dichroism (CD) Spectroscopy

CD measurements were recorded on a Jasco-815 spectropolarimeter (Japan Spectroscopic Co., Tokyo, Japan) at 25°C. The CD spectrum was monitored from 200 to 250 nm using 0.3 mg/ml protein in a 0.2 cm path length cuvette with a bandwidth of 1 nm and response time of 1 s. The data were expressed as molar ellipticity. The spectra were corrected with the respective buffer control.

For mass spectrometric analysis, purified V2 was extensively dialyzed to remove the salts and thereafter subjected to matrix-assisted laser desorption ionization-mass spectrometry analysis using a Ultraflex MALDI TOF/TOF (Bruker Daltonics) mass spectrometer equipped with a nitrogen laser (337 nm).

### Confocal microscopy

Sf21 cells were grown on coverslips and infected with recombinant baculovirus encoding GFP, GFP-V2, GFP-CP and GFP-CP NLS deletion mutant separately. 60 h of post baculoviral infection, the cells were washed with PBS and fixed with 1.5% paraformaldehyde for 30 min at RT and washed again with PBS. For direct fluorescence experiments the cells were then incubated with 1 µg/ml DAPI for 2 min., mounted with fluorescence preserver, and the samples were examined for GFP expression by confocal microscopy (Carl Zeiss LSM 510 META) and the image was processed using LSM 5 image examiner. Indirect immunofluorescence was performed by incubating the cells with respective primary and TRIT C conjugated secondary antibodies. For the colocalization analysis, the optical section of the image was chosen. Images captured at different wavelengths were superimposed, and the intensity of expression for each fluorochrome in the field was then plotted in a scatter gram. The colocalization coefficient and the correlation coefficient were obtained by LSM Colocalization software (Carl Zeiss). The background thresholds are determined by considering the optimal intensities of both red and green according to LSM localization software.

Leaf samples were examined under confocal microscopy (Carl Zeiss LSM). For detection of GFP fluorescence, excitation filter 365 nm and emission filter 420 nm were used. For YFP detection excitation filter 520 nm and emission filter 535 nm were used.

### Propidium Iodide staining

Leaf samples were stained with Propidium Iodide (PI) as described earlier [46]. The samples were fixed with PME buffer (50 mM PIPES pH 6.9, 5 mM EGTA, 2 mM MgSO_4_) containing 3% paraformaldehyde, 0.05% Triton X−100, 0.25% DMSO, 50 µM PMSF and incubated for 1 h. After the incubation, leaf samples were washed three times each for 5 min in PBS. They were then transferred to freshly prepared PI solution (final concentration 1µg/ml) in PBS and incubated for 1 h in the dark. PI solution was decanted, the leaf samples were washed four times, each for 30 min duration with PBS, dried on Whatmann #1 paper and mounted on a glass slide with anti-fading agent Elvanol to observe the fluorescence [46].

## Supporting Information

Figure S1 **GFP expression in insect cells.** The Sf21 cells expressing GFP alone was fixed and observed under the confocal microscope **(a)**. Graph plotted by quantifying the intensity of the GFP across the cell (red line) as a function of distance (in µm) further confirmed the distribution of GFP throughout the cell **(b)**.
(TIF)

Figure S2 **Multiple alignment of the deduced amino acid sequence of CLCuKV-Dab CP with representative begomoviral CP.** The crucial amino acid residues predicted by ScanProsite (http://expasy.org/tools/scanprosite/), PSORT (http://psort.nibb.ac.jp) and NetNES (http://www.cbs.dtu.dk/services/NetNES/) for putative nuclear localization signal **(A)** and nuclear export signals (NES) **(B)** of the CP sequences are shown in bold letters. Names of the viruses used for the analysis are given as abbreviations and their corresponding NCBI accession numbers are also mentioned.
(DOC)
