# Supplementary figures and images for: Functional Characterization of Coat Protein and V2 Involved in Cell to Cell Movement of Cotton Leaf Curl Kokhran Virus-Dabawali
Source: PLoS One. 2011 Nov 16;6(11):e26929. doi: 10.1371/journal.pone.0026929 (PMC3217939; doi:10.1371/journal.pone.0026929)

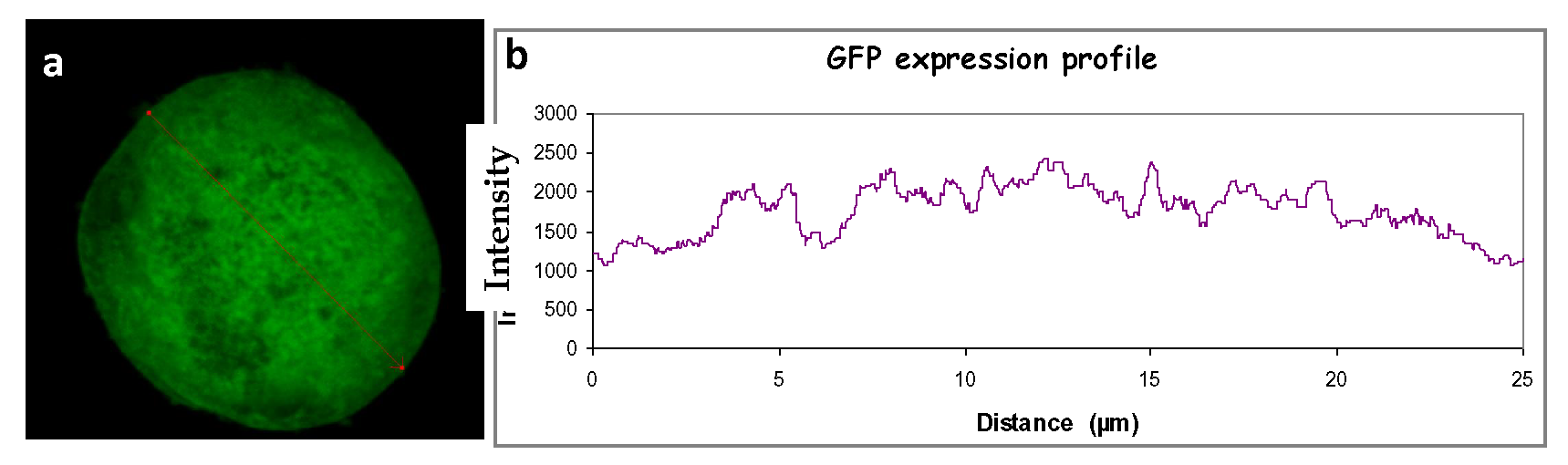

Supplement: Figure S1 — GFP expression in insect cells. The Sf21 cells expressing GFP alone was fixed and observed under the confocal microscope (a). Graph plotted by quantifying the intensity of the GFP across the cell (red line) as a function of distance (in µm) further confirmed the distribution of GFP throughout the cell (b). (TIF) [file pone.0026929.s001.tif]
