# Supplementary material for: Functional Characterization of Coat Protein and V2 Involved in Cell to Cell Movement of Cotton Leaf Curl Kokhran Virus-Dabawali
Source: PLoS One. 2011 Nov 16;6(11):e26929. doi: 10.1371/journal.pone.0026929 (PMC3217939; doi:10.1371/journal.pone.0026929)
Supplement: Figure S2 — Multiple alignment of the deduced amino acid sequence of CLCuKV-Dab CP with representative begomoviral CP. The crucial amino acid residues predicted by ScanProsite (http://expasy.org/tools/scanprosite/), PSORT (http://psort.nibb.ac.jp) and NetNES (http://www.cbs.dtu.dk/services/NetNES/) for putative nuclear localization signal (A) and nuclear export signals (NES) (B) of the CP sequences are shown in bold letters. Names of the viruses used for the analysis are given as abbreviations and their corresponding NCBI accession numbers are also mentioned. (DOC) [file pone.0026929.s002.doc]

**Supplementary figure**

**Figure S2**
